# Reply to Inzitari et al. Comment on “Blancafort Alias et al. A Multi-Domain Group-Based Intervention to Promote Physical Activity, Healthy Nutrition, and Psychological Wellbeing in Older People with Losses in Intrinsic Capacity: AMICOPE Development Study. *Int. J. Environ. Res. Public Health* 2021, *18*, 5979”

**DOI:** 10.3390/ijerph19020889

**Published:** 2022-01-14

**Authors:** Sergi Blancafort Alias, César Cuevas-Lara, Nicolás Martínez-Velilla, Fabricio Zambom-Ferraresi, Maria Eugenia Soto, Neda Tavassoli, Céline Mathieu, Eva Heras Muxella, Pablo Garibaldi, Maria Anglada, Jordi Amblàs, Sebastià Santaeugènia, Joan Carles Contel, Àlex Domingo, Antoni Salvà Casanovas

**Affiliations:** 1 Fundació Salut i Envelliment (Foundation on Health and Ageing)—UAB, Universitat Autònoma de Barcelona, 08041 Barcelona, Spain; alexander.domingo@uab.cat (À.D.); antoni.salva@uab.cat (A.S.C.); 2 Navarrabiomed, Geriatrics Department, Hospital Complex of Navarra (CHN)—Public University of Navarra (UPNA), Navarra Health Research Institute (IdisNa), 31008 Pamplona, Spain; cesar.cuevas.lara@navarra.es (C.C.-L.); nicolas.martinez.velilla@navarra.es (N.M.-V.); fabricio.zambom.ferraresi@navarra.es (F.Z.-F.); 3 Equipe Régional Vieillissement et Prévention de la Dépendance, Gérontopôle, Centre Hospitalier Universitaire de Toulouse, 31300 Toulouse, France; soto-martin.me@chu-toulouse.fr (M.E.S.); tavassoli.n@chu-toulouse.fr (N.T.); mathieu.ce@chu-toulouse.fr (C.M.); 4 Ageing and Health Department in the Andorran Healthcare System, Servei Andorrà d’Atenció Sanitaria, AD700 Escaldes-Engordany, Andorra; eheras@saas.ad (E.H.M.); pagaribaldi@saas.ad (P.G.); manglada@saas.ad (M.A.); 5 Chronic Care Program, Department of Health, Generalitat de Catalunya, 08028 Barcelona, Spain; jamblas_ext@gencat.cat (J.A.); sebastia.santaeugenia@gencat.cat (S.S.); jccontel@gencat.cat (J.C.C.); 6 Central Catalonia Chronicity Research Group (C3RG), Centre for Health and Social Care Research (CESS), University of Vic/Central University of Catalonia (UVIC-UCC), 08500 Vic, Spain

This is a reply to the comment by Inzitari et al. [1] on our recently published work about the development of AMICOPE multi-domain group-based intervention [2]. In this comment, the authors report the characteristics of +AGIL Barcelona, a multifactorial intervention program for older community-dwellers based on the integration of primary care, geriatric medicine, and community services [3]. We read the comment with interest and deeply appreciate their contributions.

Inzitari et al. state that although both +AGIL and AMICOPE intervention strategies are similar, “the design of AMICOPE seems quite selective in the inclusion of participants because it apparently excludes older adults with cognitive impairment or sensorial deficits”. Certainly, AMICOPE focuses on a phase prior to frailty with a lower prevalence of diseases and disability and with no losses in the sensorial domain, and our target population corresponds to older people without cognitive decline, severe visual impairment, or hearing loss. AMICOPE includes a session dedicated to cognitive stimulation, which can be beneficial for those participants without or with initial phases of cognitive decline. However, recent guidelines recommend specific interventions for people in later phases of cognitive decline [4], which is beyond the scope of our current approach.

Regarding the sensorial domain, although people with visual deficiency and hearing loss could undoubtably benefit from group-based multi-domain interventions, the current version of AMICOPE is not yet adapted to severe impairments in these domains, as it could limit or prevent the participation in group dynamics. We would certainly like to be able to carry out this adaptation in the future and thus would appreciate learning how this issue has been addressed in +AGIL.

Inzitari et al. also state that “+AGIL Barcelona was designed to be more adaptable to the needs of older adults and primary care staff and includes a possible more heterogeneous population”. We disagree on that statement for several reasons. Firstly, the physical activity domain of AMICOPE includes prescription passports for participants tailored to their individual functional capacity, which are assessed by the short physical performance battery, a walking speed test, and the risk of falls [5]. Secondly, AMICOPE uses goal setting as a strategy that assists individuals in identifying specific behaviors to modify, which has been considered a fundamental component of successful interventions [6]. SMART goals are mental representations of desired outcomes and thus reflect individuals’ needs, preferences, and expectations [7]. Thirdly, outdoor activities included in AMICOPE are chosen and agreed upon after a group mapping activity of local community assets. This allows each intervention to be adapted to a specific geographical context with all its specificities [8]. Finally, AMICOPE was developed in the context of the cross-border APTITUDE project, which involves 11 different territories from Occitania, Andorra, Navarra, and Catalonia. The intervention has been designed from the outset to be implemented in a wide range of rural and urban settings belonging to these territories and consequently it has the potential to reach a widely heterogeneous population from a demographic, socioeconomic, and cultural point of view [9]. Inzitari et al. state that “AMICOPE seems to have been designed to more specifically act on the promotion of mental and psychological well-being”. In that sense, we believe it is worth mentioning that a third of our intervention (10 out of 30 h) is dedicated to providing multimodal exercise through the Vivifrail program, whereas 6.5 h are dedicated to both dietary advice and to the psychological well-being component. AMICOPE was designed as a group-based approach that is complementary to those medical or social interventions that have already demonstrated efficacy in the prevention of dependency in older people: physical activity, healthy eating, and psychosocial aspects that can help increase the social capital of older people and aid them in adopting behavioral changes in accordance with the needs and preferences of pre-frail or frail older people.

According to the process of developing complex intervention [10], we are currently undertaking a feasibility study to assess the pilot implementation of AMICOPE in Andorra, which was approved by the Ethics Committee of Clinical Research at the Hospital Nostra Senyora de Meritxell. After that, the effectiveness of the refined intervention will be assessed in a randomized clinical trial funded by the Spanish Ministry of Science and Innovation [11]. 

We congratulate Inzitari et al. for the preliminary results of their intervention, which showed a very high adherence to the program and a positive pre–post impact of the intervention [3]. We look forward to how +AGIL will evolve and completely agree on the need to develop synergies and collaborative research comparing or merging data in the future. 
